# Clinical outcomes and failure rates of combined ‘Over‐the‐Top’ anterior cruciate ligament reconstruction and lateral extra‐articular tenodesis in a cohort of patients with an average age of 60 years

**DOI:** 10.1002/jeo2.70889

**Published:** 2026-08-17

**Authors:** Lorenzo Bini, Gian Andrea Lucidi, Piero Agostinone, Cristiano Liao, Alberto Grassi, Stefano Zaffagnini

**Affiliations:** ^1^ Department of Biomedical and Neuromotor Sciences DIBINEM University of Bologna Bologna Italy; ^2^ 2nd Orthopaedic and Traumatologic Clinic IRCCS Rizzoli Orthopaedic Institute Bologna Italy

**Keywords:** 60 years old, ACL, late adulthood, LET

## Abstract

**Purpose:**

The management of anterior cruciate ligament (ACL) tears in older adults remains controversial, although recent evidence suggests an increasing trend toward surgical treatment. To date, no studies have specifically investigated the outcomes of combined ACL reconstruction (ACLR) with over‐the‐top (OTT) technique and lateral extra‐articular tenodesis (LET) in this population. The aim of this study was to evaluate clinical outcomes and failure rates of ACLR using OTT plus LET in patients aged 55 years or older.

**Methods:**

Patients aged ≥55 years who underwent primary ACLR with OTT and LET techniques and had a minimum follow‐up of 2 years were included. Clinical outcomes were assessed using clinical scores, such as VAS, Lysholm, KOOS, comparing preoperative and post‐operative values. Patient Acceptable Symptom State (PASS) thresholds were applied to KOOS subscales. Reoperations and graft failures were also recorded.

**Results:**

Twenty‐eight patients met the inclusion criteria, with a mean age of 60.1 ± 3.2 years and a mean follow‐up of 6.5 ± 3.8 years. One graft failure (3.6%) and one reoperation (3.6%) were observed. Significant improvements were noted in all clinical scores. The mean Lysholm score increased from 55.4 ± 15.9 preoperatively to 96.6 ± 6.6 postoperatively. KOOS subscales showed marked improvement, particularly in Quality of Life (41.6 ± 20.2 to 90.3 ± 12.9) and Pain (68.7 ± 12.1 to 94.2 ± 5.7).

**Conclusion:**

ACLR with OTT and LET techniques in older patients demonstrated low failure rates and favourable clinical outcomes. Larger studies are needed to confirm these findings and to establish the role of this combined technique in this population.

**Level of Evidence:**

Level IV.

**Abbreviations**
ACL: anterior cruciate ligament
ACLR: anterior cruciate ligament reconstruction
ADL: activity of daily living
BMI: body mass index
IQR: interquartile range
ITB: ileo‐tibial band
KOOS: Knee injury and Osteoarthritis Outcome Score
LCL: lateral collateral ligament
LET: lateral extra‐articular tenodesis
MCL: medial collateral ligament
MRI: magnetic resonance imaging
OA: osteoarthritis
OTT: over the top
PASS: Patient Acceptable Symptom State
PCL: posterior cruciate ligament
PROMs: patient‐reported outcome measures
QoL: quality of life
ROM: range of motion
TKA: total knee replacement
UKA: unicompartimental knee replacement
VAS: Visual Analog Scale

## INTRODUCTION

Anterior cruciate ligament (ACL) reconstruction has traditionally been reserved for young and active individuals, while conservative management is generally preferred in older patients with reduced functional demands [23]. However, as participation in recreational sports continue to increase, along with life expectancy, a growing number of older patients present with symptomatic ACL tears and expectations of returning to their previous activity level [11]. In carefully selected cases, ACL reconstruction in this age group has shown satisfactory results, challenging the belief that age alone should represent a contraindication to surgery [20].

However, the indication of ACL reconstruction (ACLR) in older patients still remains considered ‘borderline’, and the potential role of additional lateral procedures such as lateral extra‐articular tenodesis (LET) has never been specifically explored in this subgroup of patients.

LET is typically indicated in young, high‐demand athletes, in case of explosive Pivot Shift or in revision surgery, aiming to improve rotational stability and reduce graft failure risk [7, 17]. Extending its indication to patients in their sixties could therefore be considered as an aggressive approach, raising concerns about an increased risk complications such as stiffness, cyclops lesion, over constraint, or accelerated osteoarthritis (OA) progression [5, 31, 32, 34]. However, adding a LET to a hamstring ACL reconstruction in this population could provide the same biomechanical advantages, such as decreased graft stress and reduced failure rate, observed in younger high‐risk patients [3, 15]. Moreover, LET addition may enhance graft protection in patients with reduced hamstring graft diameter due to age [30, 35]. Whether these theoretical benefits translate into clinical improvement in older patients remains unknown, as no previous study has specifically investigated this topic.

The purpose of this study was to analyse the clinical outcomes of a cohort of patients with an average age of 60 years old who underwent ACL reconstruction with hamstring graft and an associated LET, which was routinely performed in every patient as part of the surgical technique. The study also aimed to evaluate reoperation and failure rates and to provide a descriptive overview of the mid‐term clinical outcomes in this unconventional indication.

We hypothesised that combined ACL reconstruction and LET in older patients may provide satisfactory functional outcomes and low failure rates, without increasing the risk of post‐operative complications.

## METHODS

### Ethics

The study was approved by the institutional review board of Istituto Ortopedico Rizzoli, Bologna (Prot gen 0007875) and each patient gave informed consent to be included in the present study.

### Patient selection

A retrospective analysis was performed on patients who underwent primary OTT ACLR with hamstring tendons and LET at a single institution between 2008 and 2023, who were at least 55 years old at the time of surgery, following the age criteria adopted by Ogunleye et al. [22]. Patients were screened for eligibility applying inclusion and exclusion criteria summarised in Table 1. Knee antero‐posterior and lateral radiographs were routinely performed in order to exclude advanced OA.

**Table 1:** Inclusion and exclusion criteria.

Inclusion criteria
55 years old or more
Minimum 2 years follow‐up
Primary ACL reconstruction with OTT + LET technique and single‐bundle hamstring tendons
Exclusion criteria
Previous ipsilateral limb surgery
Primary ACL reconstruction performed with different techniques than OTT + LET technique and single‐bundle hamstring tendons
Revision ACL
Associated ligament (PCL, MCL, LCL) lesions or bone (fracture, avulsion) lesions
Additional associated surgical procedures other than meniscal repair or meniscectomies (e.g.
Meniscus transplant, cartilage procedures…)
Advanced knee osteoarthritis (Kellgren–Lawrence III–IV)

Abbreviations: LCL, lateral collateral ligament; LET, lateral extra‐articular tenodesis; MCL, medial collateral ligament; PCL, posterior cruciate ligament; OTT, over‐the‐top.

After inclusion in the study, demographic and clinical data were retrospectively collected. Information included age, sex, smoking habits, presence of diabetes, body mass index (BMI), and the time interval between injury and surgery. Surgical reports were reviewed to identify concomitant meniscal or cartilage lesions and any associated procedures. Patients who underwent additional procedures, besides meniscal treatments, were considered not eligible for inclusion.

### Patients evaluation

Eligible patients were contacted by one of the authors and asked to complete the following questionnaires: Visual Analog Scale (VAS) for pain (both at rest and during activity) [25], Lysholm score [16], and Knee injury and Osteoarthritis Outcome Score (KOOS) [26]. Pre‐operative values were collected pre‐operatively. For each KOOS subscale, patients were categorised as passing or not passing the threshold of the Patient Acceptable Symptom State (PASS) according to Muller et al. [21] (Pain, 88.9; Symptoms, 57.1; Activities of Daily Living (ADL), 100.0; Sport, 75.0; Quality of life (QoL), 62.5).

Patients reported their type of sport, frequency of activity, and Tegner activity level before injury, before surgery, and at the final follow‐up [28]. Post‐operative Tegner score was defined as the highest level reached after ACL reconstruction during the follow‐up period. Patients were also asked whether they were satisfied with the surgery and whether they would undergo the same procedure again.

Patients were also questioned regarding post‐operative complications, further knee surgeries, and any clinical or radiological evidence of graft re‐rupture. Medical records were reviewed to verify the information and to identify any reoperations, including hardware removal or additional meniscal procedures.

Conversion to unicompartmental or total knee arthroplasty (UKA or TKA), revision ACL surgery and recurrence of instability with clinical or radiological evidence of ACL re‐rupture were considered failures. Failures were excluded from PROMS calculation.

### Surgical procedure

**Figure 1:**
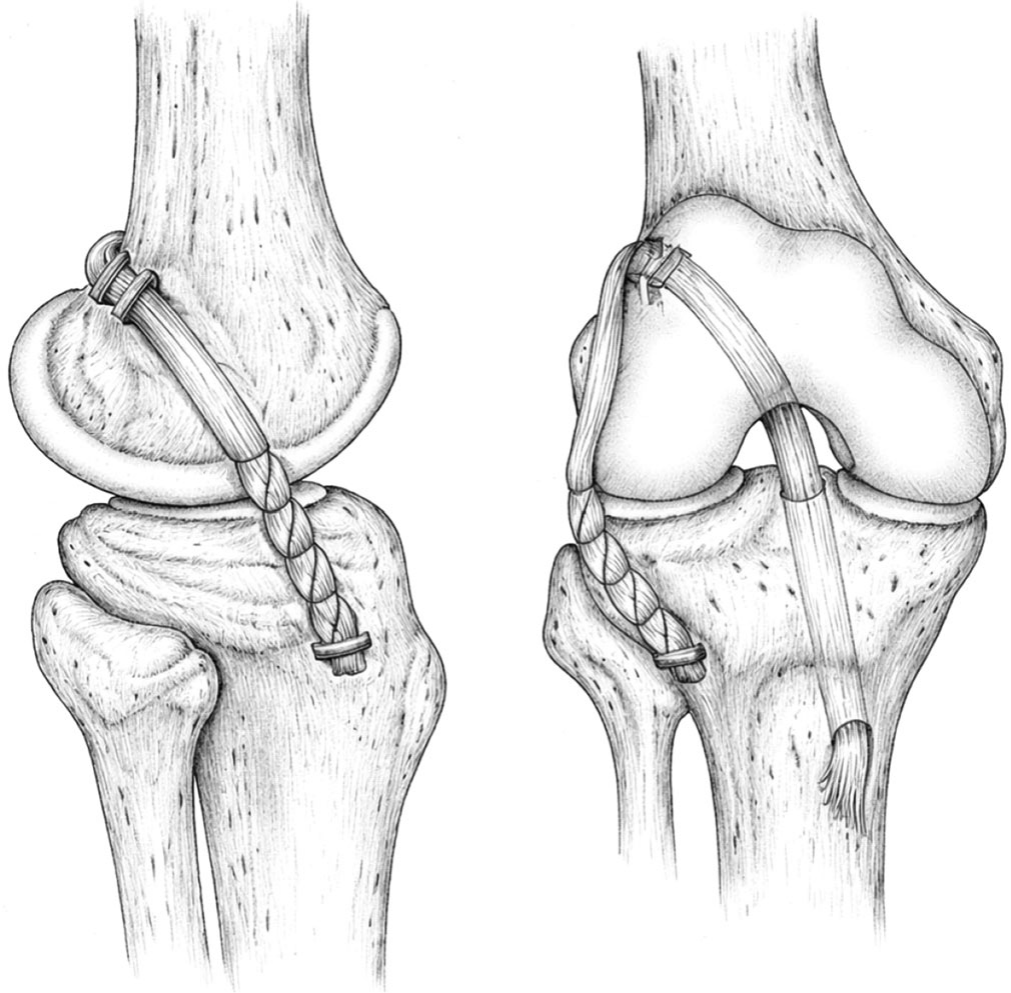
The OTT and LET ACLR technique. ACLR, anterior cruciate ligament reconstruction; LET, lateral extra‐articular tenodesis; OTT, over‐the‐top.

The same surgical technique was routinely performed in every patient [18]. After diagnostic arthroscopy, during which meniscal treatments were carried out, if needed, a single‐bundle over‐the‐top ACL reconstruction and lateral extra‐articular reconstruction using hamstring tendon autograft was performed. The gracilis and semitendinosus tendons were harvested preserving their distal insertion. Then the tibial tunnel was drilled, aiming at the postero‐medial tibial ACL footprint. Tunnel size was decided based on graft diameter after harvesting. The graft was passed through the tibial tunnel and then routed through the intercondylar notch and around the lateral femoral condyle through a lateral incision of the ileo‐tibial band (ITB) and fixed in the over‐the‐top position with two 8 mm staples; With the residual graft, the lateral tenodesis was performed, passing it deep to the ITB and superficial to the lateral collateral ligament, and fixing it on the Gerdy's tubercle with a 6 mm staple. The final configuration is displayed in Figure 1. All patients started post‐operative rehabilitation with the same protocol the day after surgery. Free range of motion exercises were started on the first day after surgery. Patients were maintained on a restricted weightbearing until the fourth week, while performing proprioceptive exercises, isometric muscle strengthening and active mobilisation exercises. From the second month patients were allowed progressive to full weight bearing. From the third month patients were allowed functional rehabilitation. Return to sport was allowed after 6 months from the surgery [33].

### Statistical analysis

Statistical analysis was performed using JASP software (version 0.19, University of Amsterdam, Amsterdam, The Netherlands). Continuous variables are presented as mean and standard deviation (SD), except for the Tegner activity level, which showed a non‐normal distribution and is therefore reported as median with interquartile range (IQR). Categorical variables are reported as absolute frequencies and proportions.

The normality of continuous variables was assessed using the Shapiro–Wilk test. For normally distributed variables, pre‐ and post‐operative scores were compared using the paired samples *t*‐test. For variables that did not meet the assumption of normality, the Wilcoxon signed‐rank test was applied. A *p*‐value of less than 0.05 was considered statistically significant. To account for multiple comparisons across the eight outcome measures, a Bonferroni correction was applied, yielding an adjusted significance threshold of *p* < 0.006. Effect sizes were calculated using Cohen's *d*, with values of 0.2, 0.5, and 0.8 considered as thresholds for small, medium, and large effects, respectively. The 95% confidence intervals (CI) of the mean pre‐to‐post‐operative differences were computed for all continuous outcome measures. For each KOOS subscale, patients were categorised according to the Patient Acceptable Symptom State (PASS) thresholds defined by Muller et al. [21], and the proportion of patients exceeding each threshold was reported.

## RESULTS

**Figure 2:**
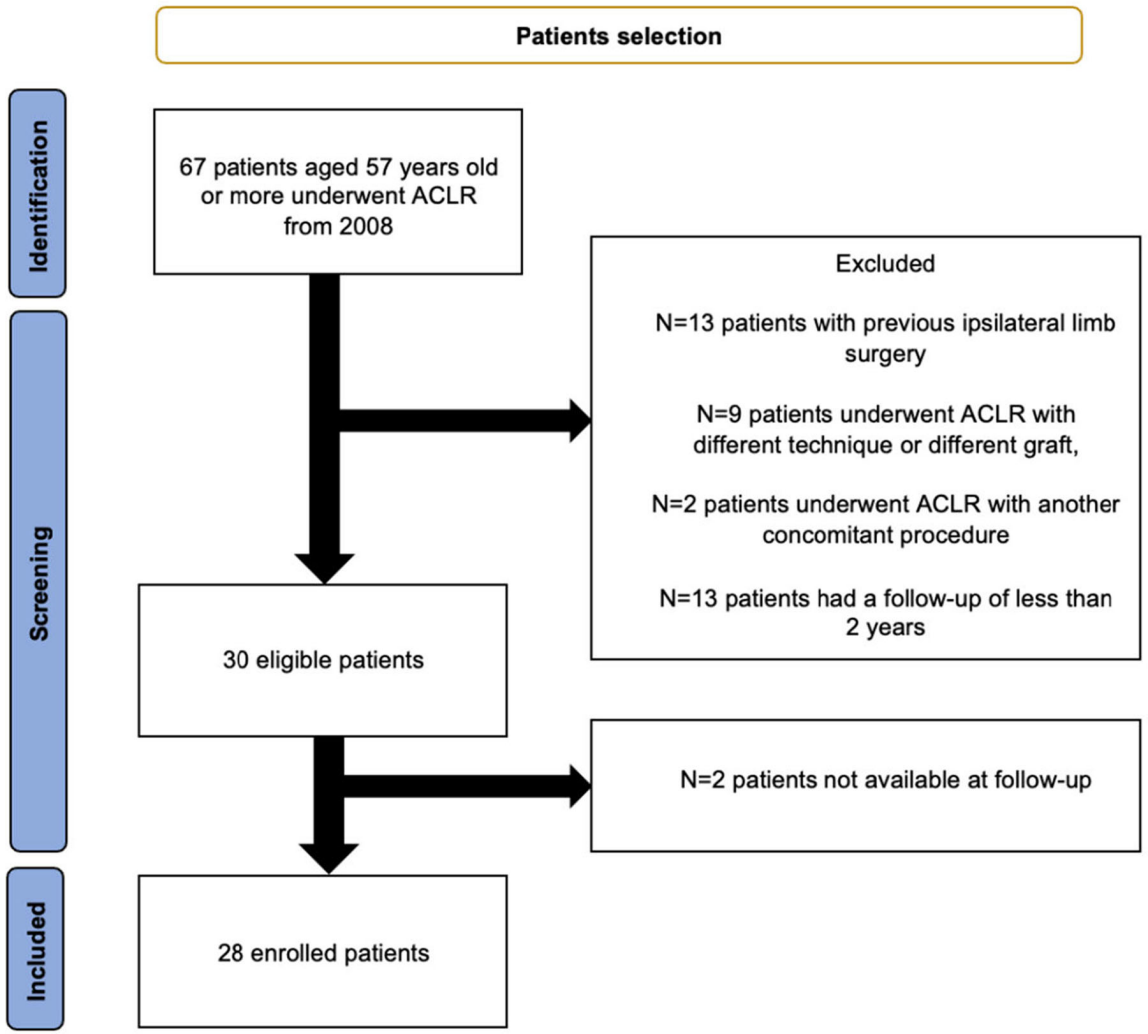
Flowchart of patients selection based on inclusion and exclusion criteria. ACLR, anterior cruciate ligament reconstruction.

A total of 30 patients met the inclusion criteria, 28 patients (93.3%) were available at follow‐up, 17 patients (60.7%) were male. Patients selection based on inclusion and exclusion criteria is reported in Figure 2.

The mean age at surgery was 60.1 ± 3.2 years. The mean follow‐up was 6.5 ± 3.8 years. A total of 23 patients (82.1%) presented concomitant meniscal lesion: 16 patients (57.1%) presented an isolated medial meniscus lesion, five patients (17.9%) had an isolated lateral meniscal lesion, while two patients (7.1%) presented both medial and lateral meniscus tears. The final graft diameter was usually 7 or 8 mm. Patients' demographics and meniscal procedures are summarised in Table 2.

**Table 2:** Patient' demographics and concomitant meniscal procedures.

Characteristics		Number of patients (%)
Sex		
Male		17 (60.7%)
Female		11 (39.3%)
Smoke		7 (25%)
Diabetes		1 (3.6%)
BMI		25.4 ± 3.9
Time between injury and surgery (months)		16.9 ± 27.5
Medial meniscus lesion *n* = 18	Meniscectomy	15 (53.6%)
	Repair	3 (10.7%)
Lateral meniscus lesion *n* = 7	Meniscectomy	4 (14.3%)
	Repair	3 (10.7%)

*Note*: Percentages reported are based on the total number of included patients in the study.

Abbreviation: BMI, body mass index.

### Patients reported outcome measures

The mean VAS score before surgery was 2.3 ± 1.6 at rest and 4 ± 1.8 during activity. At follow‐up the mean VAS was 0.3 ± 0.6 at rest and 0.7 ± 0.9 during activity. The mean Lysholm score improved from 55.4 ± 15.9 before surgery to 96.6 ± 6.6 after surgery.

The mean values of the KOOS subscales before surgery were 68.7 ± 12.1 for pain, 66.9 ± 10.6 for symptoms, 73.8 ± 11.5 for ADL, 40.5±15.9 for sports and 41.6 ± 20.2 for QoL.

At follow‐up the mean KOOS subscales improved to 94.2 ± 5.7 for pain, 91.7 ± 8.1 for symptoms, 96.8 ± 4.5 for ADL, 83.9 ± 11.1 for sports and 90.3 ± 12.9 for QoL. Effect sizes and 95% confidence intervals are reported in Table 3.

**Table 3:** Effect sizes and 95% CIs of pre‐to‐post‐operative differences.

Variable	Mean difference	95% CI	Cohen's *d*	*p*‐value
VAS rest	−2.04	[−2.71, −1.36]	1.66	<0.05
VAS activity	−3.32	[−4.11, −2.53]	2.31	<0.05
Lysholm	+41.14	[+34.47, +47.81]	3.38	<0.05
KOOS Pain	+25.53	[+20.35, +30.72]	2.70	<0.05
KOOS symptoms	+24.75	[+19.56, +29.94]	2.62	<0.05
KOOS ADL	+23.00	[+18.23, +27.77]	2.64	<0.05
KOOS Sport	+43.32	[+35.82, +50.83]	3.17	<0.05
KOOS QoL	+48.68	[+39.38, +57.98]	2.87	<0.05

*Note*: Effect sizes calculated using Cohen's *d*.

Abbreviations: CI, confidence intervals; KOOS, Knee injury and Osteoarthritis Outcome Score; KOOS ADL, KOOS Activities of Daily Living; KOOS QoL, KOOS Quality of Life; VAS, Visual Analog Scale.

Patients reported a median Tegner of 6 [5, 6] before the injury, 2 [2, 3] before surgery and 5 [4, 5, 6] at follow‐up.

PASS thresholds were applied to KOOS subscales, according to Muller et al. [21]. For KOOS pain 25 patients (89.3%) passed the threshold, for KOOS symptoms 27 patients (96.4%), 16 patients (57.1%) for KOOS ADL, 23 patients (82.1%) for KOOS sports and finally 26 patients (92.9%) for QoL. When asked about the surgery, 25 patients (89.3%) stated they would undergo surgery again, if needed, while the remaining three patients (10.7%) stated they would not.

### Return to sport

Patients were also questioned about their return to sport. 24 patients (85.7%) returned to playing sports, after a mean stop of 11.5 ± 6 months, with 17 (60.7%) going back to the same sport practiced before the injury.

### Failures and complications

**Figure 3:**
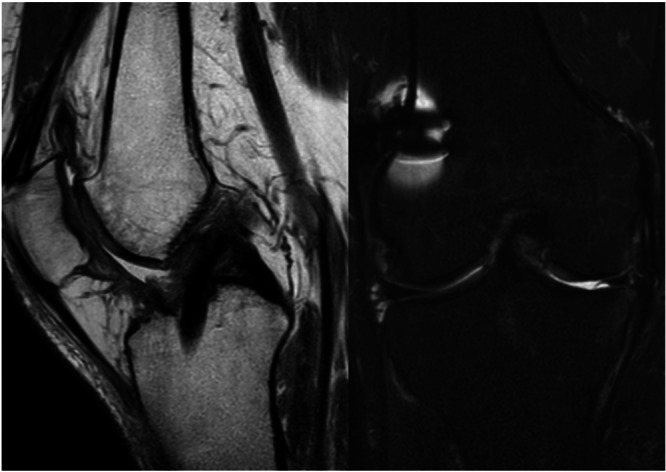
Sagittal (left) and coronal (right) MRI of a 63‐year‐old patients 3 years after ACL reconstruction with hamstring ‘Over‐the‐Top’ and associated 'Lateral‐Extra‐Articular tenodesis' and medial meniscus bucket‐handle repair. ACL, anterior cruciate ligament; MRI, magnetic resonance imaging.

Only one failure (3.6%) was recorded, due to graft failure at 36 months after surgery. The patient decided not to get revision surgery; one case (3.6%) of post‐operative stiffness was recorded. The patient underwent an arthroscopic arthrolysis after 19 months which successfully restored the knee full range of motion (ROM). No other complication or reoperation was reported during the follow‐up period. 3 years follow‐up MRI of a 63‐year‐old patient is reported in Figure 3.

## DISCUSSION

The most important finding of the present study is that ACL reconstruction with hamstring tendon using the OTT and associated LET resulted in good clinical outcomes both in terms of patients satisfaction and return to sport rate, with a low percentage of complication or revision.

The present research contributes to expand the limited body of literature on the outcomes of ACL reconstruction in a population of patients older than 60 years. Although isolated clinical series have reported encouraging outcomes following operative ACL treatment in this age group, the indication for surgical management of ACL tears in older patients remains controversial. Moreover, additional variables, such as graft type, fixation methods, and associated procedures including LET, have been poorly investigated. Even though conservative management could be proposed as definitive treatment for ACL injury in older age groups, specific factors such as subjective and objective instability, associated meniscal tears, OA grade and post‐injury lifestyle goals should be considered in the decision making process [1, 12, 13].

In a retrospective case‐series of 12 patients with a mean age of 61 years with 4.1 years of follow‐up, Toanen et al. [29] reported satisfactory PROMs on functional recovery and no increased risk of knee OA after isolated ACL reconstruction. Similarly, a retrospective study of 20 patients over 60 years of age who underwent ACL reconstruction with allograft reported overall improvement in the clinical scores but a slightly higher reoperation rate (10%), however, the follow‐up of this study was limited to one year [24]. Finally, a mid‐term follow‐up study of 37 patients (mean age 62 years) who underwent isolated ACL reconstruction with an autologous hamstring graft reported clinically meaningful functional improvements. Nevertheless, the authors observed a reinjury rate of 8.1%, and fewer than 40% of patients were able to resume sporting activity [4]. Although direct comparison is not possible, the observed failure rate and return‐to‐sport rate in our cohort appear encouraging.

Another controversial aspect of ACL reconstruction is the graft selection, as several factors, including patient age and activity level, have been associated with different clinical outcomes [14]. While in young patients, autografts such as hamstring, patellar tendon, or quadriceps tendon are the preferred options for primary ACL reconstruction, in older cohorts of patients, allografts and xenografts have also been proposed as viable alternative [6].

Studies on allograft in populations over 40 years of age have found a reasonable failure rate ranging from 2.6% to 8% at mid‐term follow‐up, with high patients satisfaction and the advantage of no donor site morbidity [2, 27]. However, more recent evidence has demonstrated even better outcomes when ACL reconstruction was performed with autografts in this subgroup of patients. In a short‐term evaluation of a group of patients with a mean age of 54 years, no failures were reported following ACL reconstruction using either hamstring or quadriceps tendon. Moreover, return to pivoting sports, such as skiing, was achieved in 81% of patients in the hamstring group and 69% in the quadriceps tendon group [19]. Building on a similar rationale, it is also possible that older patients might also benefit, in selected cases, of associated procedures such as LET that are usually reserved for high‐risk patients [8, 9]. Although LET has been advocated by some surgeons in young, high‐risk populations, where it has demonstrated a reduction in hamstring graft failure rates, its biomechanical role in decreasing rotational instability and reducing stress on the ACL graft could also be relevant in older patients who want to go back to high risk pivoting sports such as skiing [10]. While LET is not normally considered in older patients, fearing OA progression and over‐constraint, in our cohort no patient underwent conversion to TKA and only one patient underwent an arthroscopic arthrolysis for stiffness at a mean follow‐up of more than 6 years. Moreover, patients achieved promising clinical results: Except for KOOS ADL, whose PASS threshold of 100.0 requires a maximum score to be met, more than 80% of patients exceeded the PASS threshold for all remaining KOOS subscales, reflecting a high rate of clinically acceptable symptom relief in this selected cohort. It is true that older individuals generally show lower graft failure rates compared with younger, more active populations; however, the potential protective effect of LET on the reconstructed ligament may also be beneficial in older patients, where graft size could me more inconsistent compared to younger cohorts [30, 35]. Nevertheless, the safety and effectiveness of LET in this age group remain to be fully established and should be further investigated in future studies.

The present study has some limitations that should be acknowledged while interpreting the results. First, the number of included patients was limited, which may have influenced the generalisability of the findings. Moreover, the study population represents a highly selected cohort: patients were physically active, motivated to return to sport and presented without advanced knee OA. Therefore, these results should not be generalised to the average older patient with ACL deficiency. Furthermore, the high rate of concomitant meniscal injury may have influenced functional outcomes and should be considered when interpreting the results. Moreover, a standardised clinical and radiological follow‐up was not available, therefore it was not possible to evaluate objective residual laxity or osteoarthritic changes over time. Given the role of LET in limiting rotational instability, and the risk of lateral OA progression, the absence of post‐operative pivot‐shift evaluation and radiological imaging should be taken in consideration as major limitation when looking at the results. Another limitation is the absence of a control group of patients undergoing isolated intra‐articular ACL reconstruction or conservative treatment, which limits the possibility of directly comparing the outcomes of different treatment strategies. Further studies with larger sample sizes, longer follow‐up, and comparative designs are needed to better clarify these aspects.

## CONCLUSION

While conservative treatment for ACL injuries is normally the first choice in older cohorts, in patients with high functional demand and return to sport expectation, surgery can be considered and ACLR with an OTT and LET technique has shown good clinical results.

With population aging and participation in sports increasing, more and more ‘older’ patients report ACL injuries and require surgery to go back to their active life. In selected patients, who don't want to give up sport and complain knee instability, the OTT and LET technique could be a viable surgical approach to regain stability, improve quality of life and go back to sports.

## AUTHOR CONTRIBUTIONS

Conceptualisation: Gian Andrea Lucidi and Stefano Zaffagnini. Methodology: Gian Andrea Lucidi and Piero Agostinone. Validation: Gian Andrea Lucidi and Piero Agostinone. Formal analysis: Gian Andrea Lucidi and Lorenzo Bini. Investigation: Lorenzo Bini and Cristiano Liao. Resources: Lorenzo Bini and Cristiano Liao. Data curation: Gian Andrea and Lucidi, Lorenzo Bini. Writing—original draft preparation: Gian Andrea Lucidi, Piero Agostinone and Lorenzo Bini. Writing—review and editing: Gian Andrea Lucidi, Piero Agostinone and Lorenzo Bini. Supervision: Gian Andrea Lucidi and Stefano Zaffagnini. Project administration: Gian Andrea Lucidi. All authors have read and agreed to the published version of the manuscript.

## FUNDING INFORMATION

The authors have no funding to report.

## CONFLICT OF INTEREST STATEMENT

Alberto Grassi: Smith & Nephew: Not paid consultant. Stefano Zaffagnini: DePuy, A Johnson & Johnson Company: Paid presenter or speaker, paid consultant; European Society of Sports Traumatology Knee Surgery and Arthroscopy (ESSKA): Board or committee member; International Society of Arthroscopy, Knee Surgery, and Orthopaedic Sports Medicine (ISAKOS): Board or committee member; Journal of Experimental Orthopaedics (JEO): Editorial or governing board; Smith & Nephew: Paid presenter or speaker, paid consultant.

## ETHICS STATEMENT

The study was approved by the institutional review board of Istituto Ortopedico Rizzoli, Bologna (Prot gen 0007875) and each patient gave informed consent to be included in the present study. All patients accepted to be included in the study.

## Data Availability

The data that support the findings of this study are available on request from the corresponding author. The data are not publicly available due to privacy or ethical restrictions.
